# Deep Fungal Stromal Keratitis After Cataract Surgery: A Case Report

**DOI:** 10.7759/cureus.83101

**Published:** 2025-04-27

**Authors:** Seika Narai, Atsuhiko Fukuto, Tai-ichiro Chikama, Yosuke Harada, Hirokazu Sakaguchi

**Affiliations:** 1 Department of Ophthalmology and Visual Sciences, Graduate School of Biomedical and Health Sciences, Hiroshima University, Hiroshima, JPN

**Keywords:** cataract surgery, corneoscleral tunnel infection, filamentous fungi, fungal keratitis, in vivo confocal microscopy

## Abstract

Fungal keratitis is a vision-threatening infection that typically originates at the ocular surface and gradually invades the deeper layers of the cornea. We report a rare fungal keratitis confined to the deep stromal layer that developed following cataract surgery. An 81-year-old woman presented with a creeping white opacity on the posterior corneal surface in the right eye. Anterior segment optical coherence tomography revealed a hyper-reflective lesion at the posterior cornea, and in vivo confocal microscopy showed filamentous structures in the deep stroma, supporting the diagnosis of fungal keratitis. Corneal scraping was not feasible because the lesion was deep within the cornea. Initial treatment with natamycin and fluconazole was ineffective, and the infection progressed despite topical and systemic voriconazole. Ultimately, the infection resolved after administering amphotericin B via topical, intrastromal, and intravenous routes. The patient’s condition improved without requiring therapeutic keratoplasty. This case illustrates the diagnostic value of in vivo confocal microscopy in deep stromal fungal keratitis. It emphasizes the importance of selecting appropriate antifungal agents when first-line treatments are ineffective.

## Introduction

Fungal keratitis is a severe ocular infection that can cause irreversible vision loss if not promptly treated [1]. It originates at the ocular surface and gradually extends into the deeper corneal layers [2]. Cases of fungal keratitis confined solely to the deep corneal stroma are rare and pose diagnostic challenges, as corneal scraping often fails to identify the causative organism [3]. Here, we report a rare fungal keratitis localized to the deep corneal stroma that developed following cataract surgery.

## Case presentation

**Figure 1:**
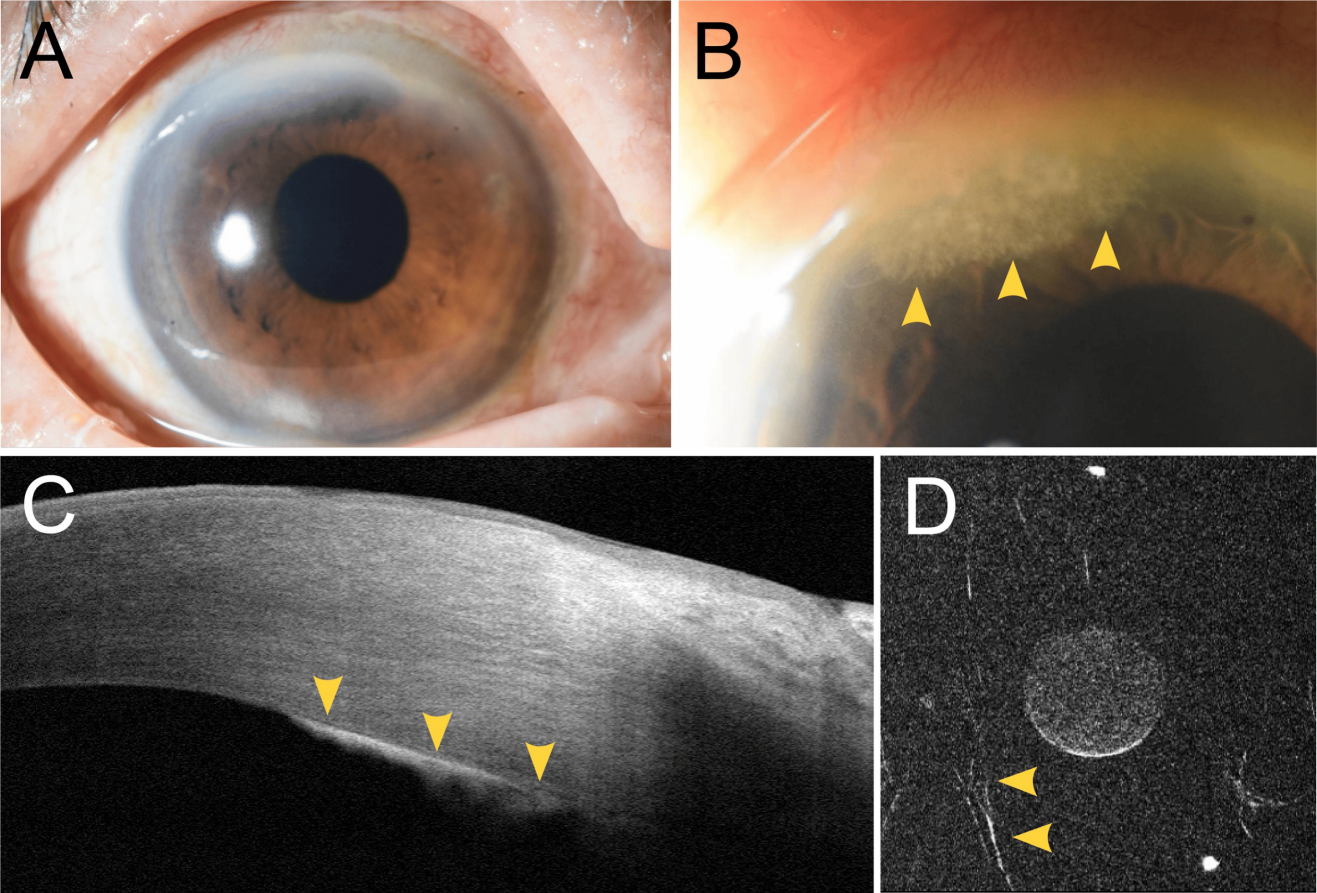
Anterior segment findings of the right eye. (A) Diffuse illumination. (B) Scleral scatter illumination revealed a posterior corneal opacity (yellow arrowheads). (C) Anterior segment optical coherence tomography (AS-OCT) image of the superior cornea revealed a hyper-reflective lesion at the posterior corneal surface (yellow arrowheads). (D) In vivo confocal microscopy of the deep corneal stroma revealed filamentous structures (yellow arrowheads).

The patient was an 81-year-old woman with a medical history of hypertension, diabetes mellitus, and bilateral dacryocystitis. She underwent bilateral cataract surgery at a local ophthalmology clinic. Two months later, conjunctival injection and anterior chamber inflammation were observed in her right eye, raising suspicion of endophthalmitis. Pars plana vitrectomy was subsequently performed. Culture of the vitreous sample obtained during surgery yielded negative results. Postoperatively, she was prescribed topical betamethasone phosphate 0.1%. Two months after the vitrectomy, a feathery white infiltrate suggestive of fungal infection was noted at the posterior corneal surface of the right eye, and she was referred to the Department of Ophthalmology at Hiroshima University Hospital. Her best-corrected visual acuity in the right eye at the initial visit was 20/20. There was no conjunctival hyperemia, and no inflammatory cells were observed in the anterior chamber or vitreous (Figure 1A). A white, creeping infiltrate was observed on the posterior surface of the cornea, and anterior segment optical coherence tomography revealed a corresponding hyper-reflective lesion at the posterior cornea (Figures 1B, 1C). Fungal keratitis was suspected at the initial visit, prompting immediate discontinuation of betamethasone eye drops and initiation of natamycin 5% eye drops, fluconazole 0.2% eye drops, and oral fluconazole 200 mg twice daily. Two days later, marked conjunctival hyperemia and anterior chamber inflammation developed in the right eye. In vivo confocal microscopy revealed filamentous structures, confirming the diagnosis of fungal keratitis (Figure 1D).

**Figure 2:**
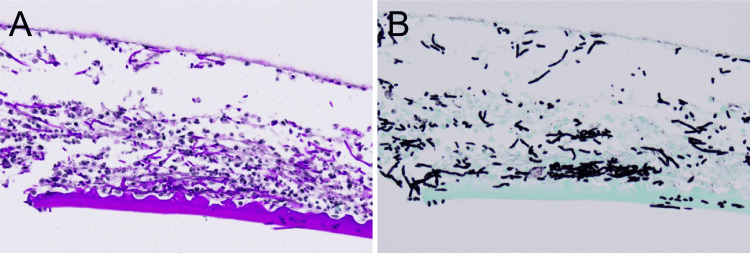
Histopathological images of the excised tissue. Top: stromal side; bottom: endothelial side. (A) Periodic acid–Schiff stain. (B) Grocott stain. All images were taken at ×20 magnification.

Due to worsening conjunctival injection and anterior chamber inflammation, fluconazole eye drops were discontinued. Voriconazole 1% eye drops (six times daily) and oral voriconazole 400 mg/day were initiated. Despite treatment, the posterior corneal infiltrate enlarged, conjunctival hyperemia worsened, and hypopyon developed. On day seven after the initial visit, anterior chamber irrigation was performed. During surgery, Descemet’s membrane at the lesion site was detached and excised. Histopathological examination of the excised tissue revealed fungal hyphae within the deep stroma and Descemet’s membrane, which stained positively with periodic acid-Schiff and Grocott stains (Figure 2).

**Figure 3:**
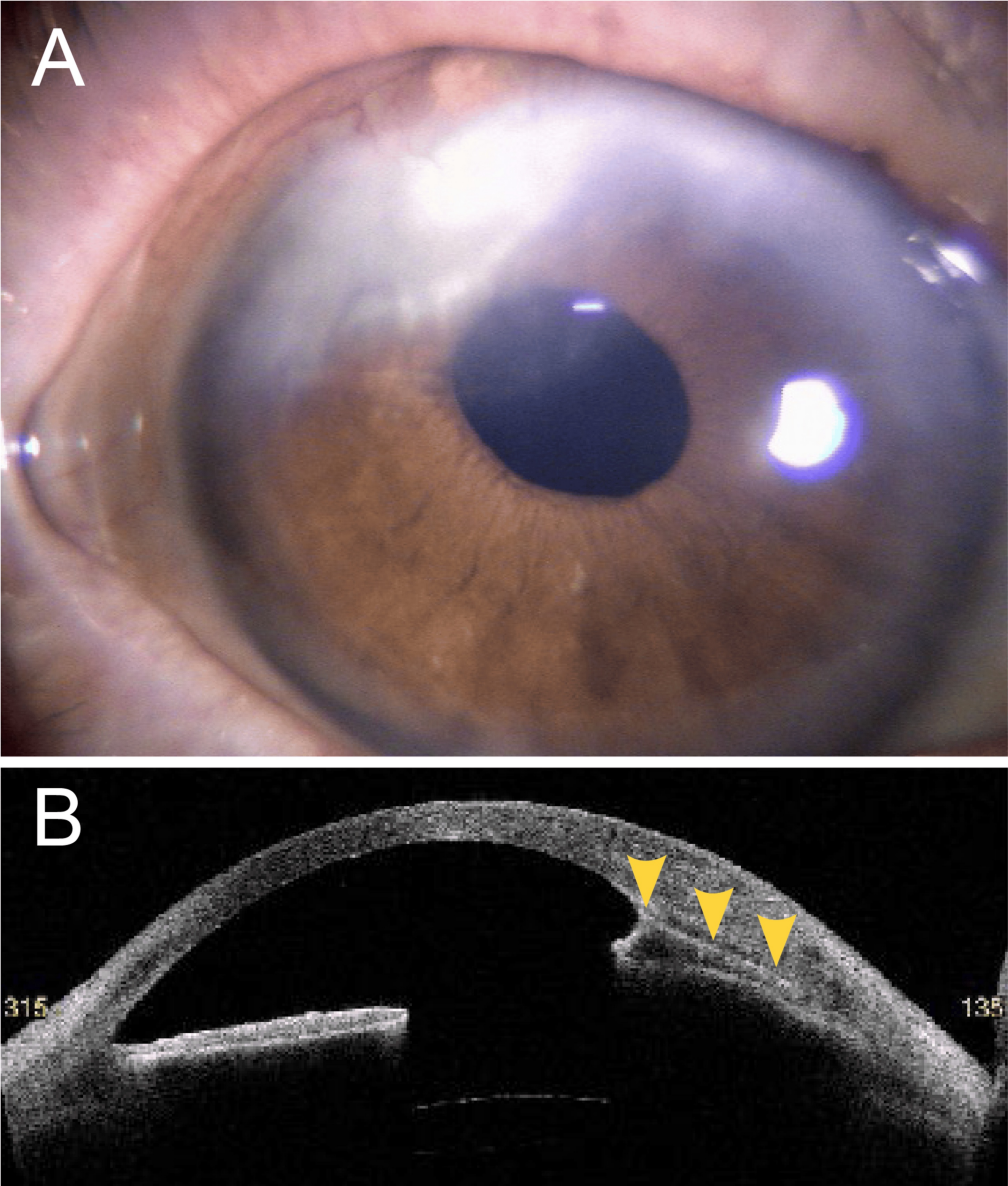
Anterior segment findings at four months after the initial visit. (A) The superior corneal lesion had healed with scarring. (B) Anterior segment optical coherence tomography (AS-OCT) shows anterior synechiae (yellow arrowheads).

Culture of the excised tissue was negative. Following surgery, intravenous voriconazole 400 mg/day, topical voriconazole 1%, and intrastromal voriconazole injections were administered. However, the infection did not improve. On day 16, a second anterior chamber irrigation was performed, and on day 20, antifungal treatment was switched to intravenous amphotericin B 120 mg/day, topical amphotericin B 0.1%, and intrastromal amphotericin B injections. Conjunctival hyperemia gradually improved, and one month after the initial visit, the posterior corneal infiltrate had resolved. Amphotericin B was then discontinued. No recurrence of fungal infection was observed during the three-month follow-up period after cessation of antifungal therapy (Figure 3).

## Discussion

The significant risk factors for fungal keratitis include soft contact lens wear, a history of corneal transplantation, ocular surface diseases, and ocular trauma [1,2]. Fungal infection occurring at or near the site of a cataract surgery incision, as observed in this case, has been infrequently documented in the literature [4-10]. Most reported pathogens have been of *Aspergillus* species [4,6,8-10], and some cases have ultimately required therapeutic keratoplasty [6-9]. Although the causative organism could not be identified in our case, antifungal therapy proved effective, allowing us to avoid corneal transplantation. There is no consensus regarding the optimal choice of antifungal agents for treating fungal keratitis. However, natamycin is generally considered the first-line treatment, and voriconazole has been reported to be effective, particularly for non-*Fusarium* infections [11]. In the present case, topical natamycin showed minimal efficacy, prompting the addition of topical and systemic voriconazole. Despite two anterior chamber irrigations, the infection relapsed. Subsequently, treatment was switched to amphotericin B via topical administration, intrastromal injection, and intravenous infusion, ultimately leading to the infection's resolution. Although amphotericin B is generally not preferred as a first- or second-line agent due to its potential for serious adverse effects such as nephrotoxicity and hypokalemia, it proved effective in this case. In vivo confocal microscopy enables real-time diagnosis of fungal keratitis and allows visualization of fungal hyphae located in the deep corneal stroma [3]. When used for the diagnosis of fungal keratitis, in vivo confocal microscopy has been reported to have a sensitivity of 94% and a specificity of 78% [12]. Corneal scraping could not be performed in the present case because the lesion was confined to the deep stroma. However, in vivo confocal microscopy facilitated early diagnosis and confidently supported the continued administration of antifungal agents.

## Conclusions

This case demonstrates that fungal keratitis can occur in the deep corneal stroma following cataract surgery, even without surface involvement. In vivo confocal microscopy, a real-time and noninvasive imaging modality, was instrumental in establishing an early diagnosis and guiding antifungal therapy when conventional scraping was not feasible. Prompt initiation of appropriate antifungal treatment, including amphotericin B, led to successful resolution without the need for keratoplasty.
